# High expression of TROP2 characterizes different cell subpopulations in androgen-sensitive and androgen-independent prostate cancer cells

**DOI:** 10.18632/oncotarget.9876

**Published:** 2016-06-07

**Authors:** Jinhan Xie, Christina Mølck, Sophie Paquet-Fifield, Lisa Butler, Australian Prostate Cancer Bioresource, Erica Sloan, Sabatino Ventura, Frédéric Hollande

**Affiliations:** ^1^ Drug Discovery Biology, Monash Institute of Pharmaceutical Sciences, Monash University, Parkville, Australia; ^2^ Department of Pathology, The University of Melbourne, Parkville, Australia; ^3^ School of Medicine, South Australian Health and Medical Research Institute, University of Adelaide, Adelaide, South Australia, Australia; ^4^ Cousins Center for PNI, UCLA Semel Institute, Jonsson Comprehensive Cancer Center, and UCLA AIDS Institute, University of California Los Angeles, Los Angeles, CA, USA; ^5^ Peter MacCallum Cancer Centre, Division of Cancer Surgery, East Melbourne, Victoria, Australia; ^6^ Current address: Children's Cancer Institute, Lowy Cancer Research Centre, University of New South Wales, Kensington, Australia

**Keywords:** prostate cancer, TROP2, self-renewal, treatment resistance

## Abstract

Progression of castration-resistant tumors is frequent in prostate cancer. Current systemic treatments for castration-resistant prostate cancer only produce modest increases in survival time and self-renewing Tumor-Initiating Cells (TICs) are suspected to play an important role in resistance to these treatments. However it remains unclear whether the same TICs display both chemo-resistance and self-renewing abilities throughout progression from early stage lesions to late, castration resistant tumors. Here, we found that treatment of mice bearing LNCaP-derived xenograft tumors with cytotoxic (docetaxel) and anti-androgen (flutamide) compounds enriched for cells that express TROP2, a putative TIC marker. Consistent with a tumor-initiating role, TROP2^high^ cells from androgen-sensitive prostate cancer cell lines displayed an enhanced ability to re-grow in culture following treatment with taxane-based chemotherapy with or without androgen blockade. TROP2 down-regulation in these cells reduced their ability to recur after treatment with docetaxel, in the presence or absence of flutamide. Accordingly, in *silico* analysis of published clinical data revealed that prostate cancer patients with poor prognosis exhibit significantly elevated TROP2 expression level compared to low-risk patients, particularly in the case of patients diagnosed with early stage tumors. In contrast, in androgen-independent prostate cancer cell lines, TROP2^high^ cells did not exhibit a differential treatment response but were characterized by their high self-renewal ability. Based on these findings we propose that high TROP2 expression identifies distinct cell sub-populations in androgen-sensitive and androgen-independent prostate tumors and that it may be a predictive biomarker for prostate cancer treatment response in androgen-sensitive tumors.

## INTRODUCTION

Prostate cancer is second to lung cancer in incidence worldwide, and is the third most common cause of cancer death in developed countries [1]. Small prostatic carcinomas can be detected in up to 29% of men aged 30 to 40 years and 64% of men aged 60-70 years [2]. Androgen deprivation is the mainstay of treatment for men with locally advanced or metastatic disease, but surviving cancer cells frequently become highly aggressive and metastatic, and resistant to further treatment [3]. A small population of prostate cancer cells with tumor-initiating cell properties has been shown to harbor intrinsic characteristics that make them resistant to androgen deprivation and chemotherapy [4, 5]. Since they also display a robust self-renewing ability and appear to drive tumor progression, their identification, characterization and elimination would provide a significant therapeutic advantage, but little is known on how the chemo-resistance and self-renewing abilities of prostate TICs evolve during progression from early androgen-sensitive lesions to late, castration resistant tumors.

Several combinations of markers have been proposed to identify prostate tumor-initiating cells. Such molecular signatures include surface expression of CD133 [6], CD49f, Sca-1 [7], CD44 [8] and the enzymatic activity of aldehyde dehydrogenase (ALDH) [9]. High expression of the cell surface glycoprotein TROP2 has been reported in sphere-forming, stem/progenitor prostate epithelial cells [10, 11], where its activity may contribute to enhance self-renewal and drive hyperplasia [12]. TROP2 is frequently upregulated in prostate carcinoma and appears to promote metastasis via the modulation of β1 integrin [13]. However, it remains unclear whether TROP2 marks tumor-initiating cells in androgen-sensitive and castration resistant prostate tumors and if it plays a role in the resistance of these cells to therapy.

To address this we investigated the expression and function of TROP2 in androgen-sensitive and -resistant prostate cancer cells. We found that TROP2^high^ tumor cells were enriched following treatment of LNCaP-derived prostate cancer xenografts. Cells with extracellular membrane expression of TROP2 displayed different properties in androgen-sensitive and androgen-independent cellular contexts. In androgen-sensitive populations TROP2^high^ cells displayed an enhanced ability to withstand treatment with high concentrations of taxane-based chemotherapy with or without androgen blockade, while in androgen-independent prostate cancer cells high TROP2 expression marked a highly self-renewing cell sub-population that showed no differential response to treatment.

TROP2 down-regulation increased the sensitivity of androgen-sensitive cells to docetaxel, in the presence or the absence of the anti-androgen compound flutamide. Analysis of published clinical data indicated that high TROP2 expression correlated with poorer recurrence-free survival in prostate cancer patients. In particular, patients with low grade (Gleason 6) tumors that expressed high TROP2 mRNA levels had significantly worse prognosis compared to those with tumors expressing low TROP2 levels. Based on these findings we propose that high TROP2 expression identifies TIC sub-populations with different phenotypes in androgen-sensitive and androgen-independent prostate tumors and that it may represent a worthwhile predictive biomarker for prostate cancer treatment response in androgen-sensitive tumors.

## RESULTS

### TROP2 expression levels correlates with disease outcome in prostate cancer patients

**Figure 1:**
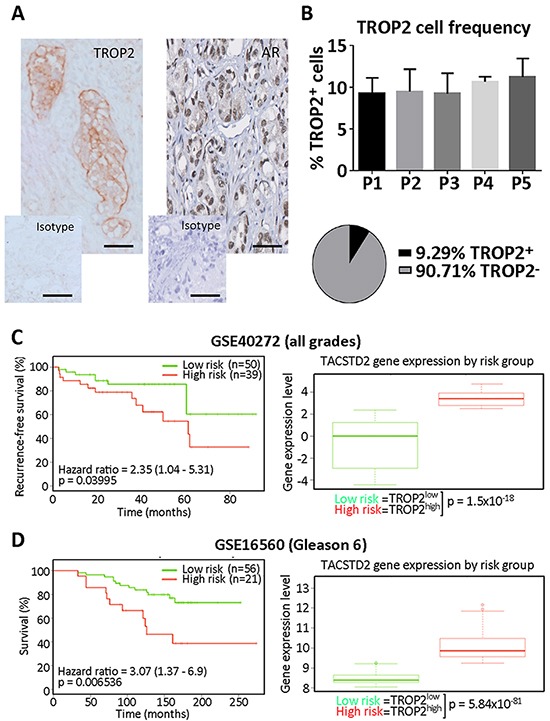
**A.** Representative microphotograph depicting tumors cells positively stained with selective antibodies against TROP2 (left) and the androgen receptor (AR, right) in a human prostate carcinoma slide, compared with the same slide stained with the isotype control antibodies. Scale bars = 50μm. **B.** Top, frequency of cells displaying TROP2 staining at the cell membrane (expressed as percent of tumor cells) in tissue sections from 5 patients (P1-5) with prostate carcinoma (P1: unknown Gleason grade; P2: Gleason >9; P3: Gleason = 9; P4: Gleason = 6; P5: Gleason = 6); Bottom, pie chart summarizing the mean percentage of TROP2 positive and TROP2 negative cells across all patients. **C.** Kaplan-Meier curve showing the recurrence-free survival (left) and box-plot showing TROP2 expression levels (right) in Low and High risk prostate cancer patient groups (Data from GSE40272, total number of samples = 89). Expression data is provided as median and 5-95 percentile in each patient group. **D.** Kaplan-Meier curve showing the overall survival (left) and box-plot showing TROP2 expression levels (right) in Low and High risk groups from patients with Gleason 6 prostate cancer (Data from GSE40272, total number of samples for this grade = 77). In C and D, hazard ratios with their confidence interval are provided (left), as well as p values for the log-rank testing equality of survival curves (left) and for the difference between TROP2 expression in low- and high-risk groups, using t-test (right). TROP2 expression data is provided as median and 5-95 percentile in each patient group.

We first analyzed the expression of TROP2 using IHC in a small subset of prostate tumors with increasing Gleason grade. Regardless of the Gleason grade, TROP2 was mostly expressed by basal layer cells of the prostate epithelium. No significant difference in TROP2 expression was detected among our samples (Figure 1A), suggesting that a minority of tumor cells express TROP2 during all stages of prostate tumor progression. In addition, we mined the SurvExpress Web resource [14] to determine whether TROP2 expression level in prostate tumors at the time of surgery was correlated with specific disease outcomes in previously published datasets. Patients with high tumor TROP2 expression displayed significantly worse recurrence-free survival than those expressing low levels of TROP2 in a small population of prostate cancer patients [15] (p=0.03995, n=89, Figure 1B). Using a larger dataset [16] we analyzed whether a similar distinction between TROP2-high and TROP2-low tumors could be detected irrespective of Gleason grading, the main current prognostic indicator in prostate cancer. We found that significantly elevated TROP2 expression characterized a subgroup with significantly worse overall survival in patients with early stage prostate tumors (Gleason 6, p=0.006536, n=77) (Figure 1C), while TROP2 levels were not significantly correlated with differences in survival for other Gleason grades (Supplementary Figure S1). Taken together, our findings suggest that TROP2-expressing cells are detectable across all prostate cancer stages but that differences of TROP2 expression levels among patients may highlight a worse prognosis for prostate cancer patients, particularly for those diagnosed with early stage tumors.

### TROP2 expression in tumors from LNCaP xenografts treated with flutamide and docetaxel

In view of the poorer prognosis exhibited by TROP2-high low-grade tumors, we aimed to determine whether TROP2 expression is associated with chemoresistance in androgen-sensitive tumor cells. To do so androgen-sensitive LNCaP cells were xenografted into NOD/SCID mice, and recurrence after treatment with docetaxel chemotherapy (10 mg/kg/week) and/or with the anti-androgen compound flutamide (33 mg/kg/day) was investigated. Treatment commenced when tumors reached 100mm^3^ and tumor volume was measured to determine the growth rate of tumors under different treatment conditions. Efficacy of docetaxel and flutamide treatments was corroborated by their respective impact on testes and prostate weight compared to control mice (Supplementary Figure S2).

**Figure 2:**
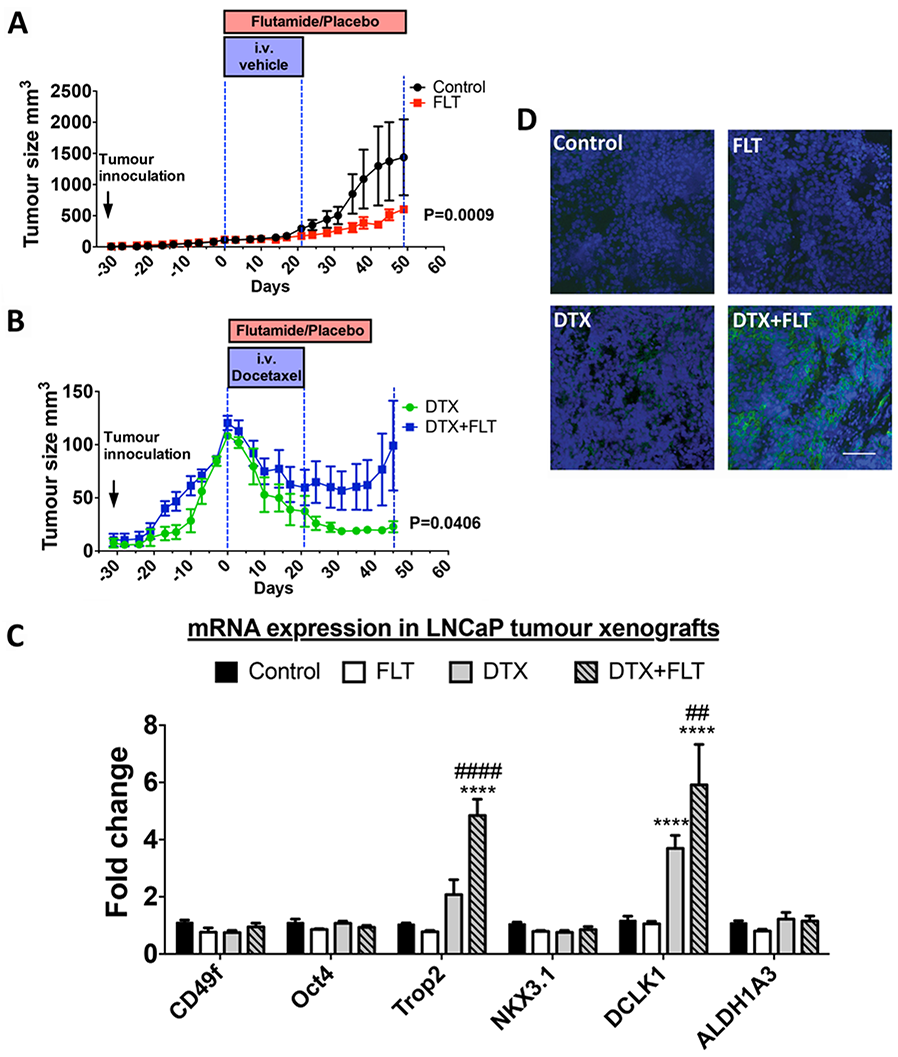
**A–B.** Tumor growth in NOD/SCID mice treated with vehicle (control), 33mg/kg/day flutamide (FLT), 10mg/kg/week docetaxel (DTX) or a combination of docetaxel and flutamide (DTX + FLT), as indicated. Data was analyzed by two-way repeated measures ANOVA and a P value < 0.05 for the interaction between treatments and time was considered to be statistically significant (n = 5 mice/group). **C.** mRNA expression levels in prostate tumors derived from LNCaP cell xenografts treated as above. *, P<0.05 and ****, P<0.0001, significantly different from control and flutamide treatment groups. ##, P<0.01 and ####, P<0.0001, significantly different from docetaxel treatment group. **D.** Immunohistochemical staining of TROP2 on tumors derived from xenografts treated as indicated in (A) and (B). Scale bar=100μm.

Flutamide treatment did not induce a regression of tumor volume but slowed down tumor growth (day 0: 114.8 ± 2.5mm^3^; day 49: 603.5 ± 60.1mm^3^) compared to the control group (day 0: 108.5±0.4 mm^3^, day 49: 1438.0 ± 609.7mm^3^) (P = 0.0009) (Figure 2A). Docetaxel alone or in combination with flutamide decreased tumor volume (Figure 2B), and post-treatment tumor recurrence was delayed in mice treated with docetaxel alone compared to those who received a combination of flutamide and docetaxel (P = 0.0406). At day 21 (end of docetaxel treatment), mice from both groups had similar tumor volumes (docetaxel: 45.6 ± 13.9mm^3^; combined: 51.8 ± 13.5mm^3^ P = 0.3512), but after docetaxel treatment had finished, tumor volume in mice treated with docetaxel alone continued to decrease (day 35: 24.9 ± 5.0mm^3^) whereas mice from the combination treatment group maintained a steady tumor size (day 35: 52.0 ± 18.6mm^3^) (P = 0.0454). RT-qPCR was used to quantify the expression of TROP2 mRNA as well as that of putative markers for basal/cancer stem cells (CD49f, POU5F1 (Oct4), DCLK1, ALDH1A3) or luminal stem cells (NKX3.1) [17] in residual tumors (Figure 2C). Expression of TROP2 and DCLK1 mRNAs was robustly up-regulated in tumors following relapse from the combination treatment of flutamide and docetaxel, while recurring tumors showed no change in CD49f, Oct4, NKX3.1 or ALDH1A3 (Figure 2C). High extracellular TROP2 expression was also detected by immunohistochemistry in tumors that underwent combination treatment (Figure 2D). These findings demonstrate that TROP2 is enriched in tumor cells that recur after treatment of androgen-sensitive xenografts with docetaxel/androgen ablation combination therapy.

### Androgen-sensitive TROP2^high^ prostate cancer cells display higher resistance to high dose chemotherapy and androgen deprivation

**Figure 3:**
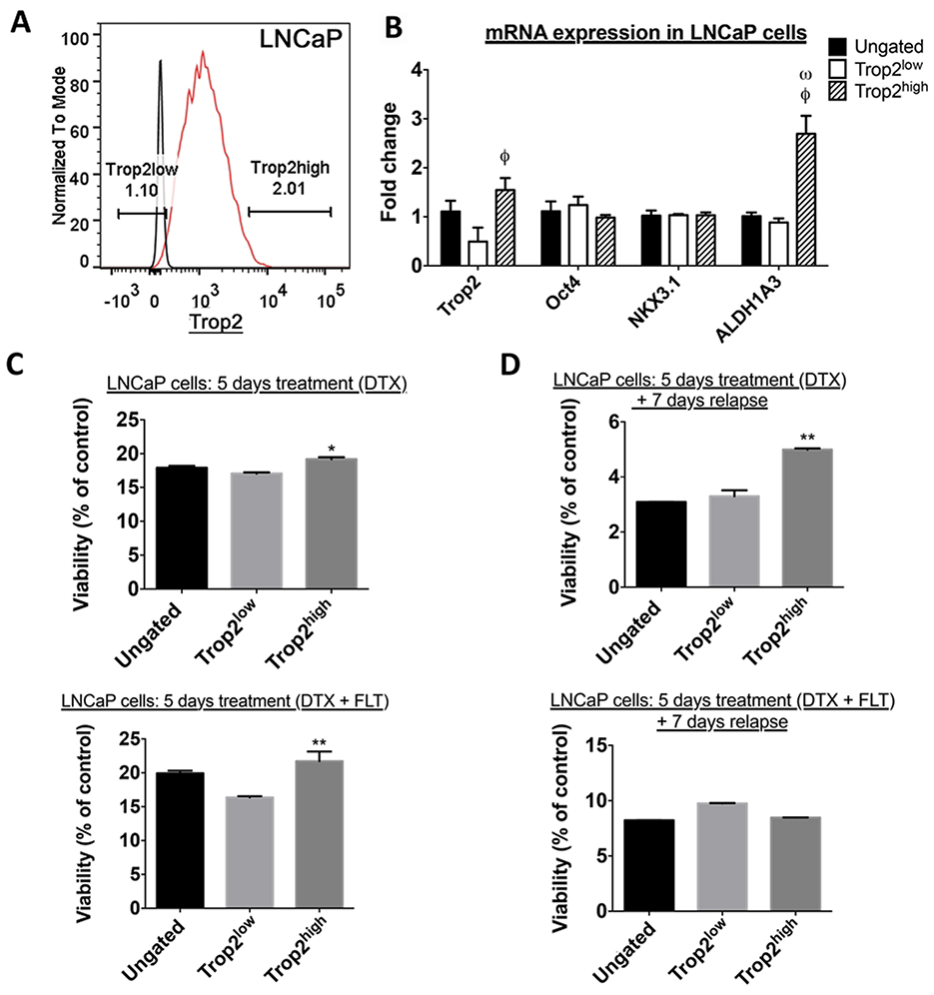
**A.** FACS profile of LNCaP cells according to their surface TROP2 expression level. Black line: cells were stained mouse isotype control; red line: cells were stained with TROP2 antibody. **B.** RT-qPCR quantitation of *TACSTD2* (TROP2), *POU5F1* (Oct4), *NKX3.1*, and *ALDH1A3* mRNA expression. *DCLK1* was undetectable in these cells **C.** Percentage of viable TROP2^high,^ TROP2^low^ and ungated LNCaP cells at the end of a 5-day treatment with docetaxel alone (DTX, top) or in combination with flutamide (DTX + FLT, bottom). Data represents the mean percentage of surviving cells after exposure to the three highest concentrations of docetaxel (1nM, 10nM, 100nM). **D.** Percentage of viable TROP2^high,^ TROP2^low^ and ungated LNCaP cells after a 5-day treatment with docetaxel alone (DTX, top) or in combination with flutamide (DTX + FLT, bottom) followed by a 7-day recovery phase in docetaxel-free medium. Data represents the mean percentage of surviving cells after exposure to the three highest concentrations of docetaxel (1nM, 10nM, 100nM). In C and D: *, P < 0.05, **, P < 0.01, significantly different from TROP2^low^ cells, one-way ANOVA with Bonferroni post-hoc, n=3.

Because Tumor-Initiating Cells (TICs) are thought to play a role in enhanced treatment resistance, we then investigated whether cells demonstrating high levels of membrane TROP2 expression exhibit TIC properties in androgen-sensitive human prostate cancer cells. LNCaP cells that highly expressed extracellular TROP2 did not stand out as a separate population. Instead, cells presented with a continuum of low to high extracellular TROP2 levels, and the top 2% of cells with the brightest fluorescence after labeling with a selective antibody were chosen as TROP2^high^ cells (Figure 3A). Baseline growth was similar in TROP2^high,^ TROP2^low^ and ungated LNCaP cells (Supplementary Figure S2). Extreme Limiting Dilution Analysis showed that there was no difference in sphere-forming efficiency (Table 1 and Supplementary Figure S2) between TROP2^high,^ TROP2^low^ and ungated LNCaP cells. This result indicates that TROP2^high^ cells do not display enhanced self-renewal abilities compared to other cell populations, despite the mRNA enrichment for the putative tumor-initiating cell marker ALDH1A3 in TROP2^high^ cells (Figure 3B). Similarly, no significant difference was detected between the sphere-forming efficiency of TROP2^high^ and TROP2^low^ and ungated cells from another androgen-sensitive cell line, 22Rv1, as determined using ELDA (Table 1 and Supplementary Figure S2). These results suggest that High TROP2 expression does not selectively enrich pre-existing cell populations with high self-renewal ability in androgen-sensitive prostate cancer cells.

**Table 1:** Sphere-forming efficiency of LNCaP and 22Rv1 cells sorted based on their extracellular Trop2 expression level, as calculated using the ELDA webtool

Cell population	Sphere-forming frequency of androgen-sensitive prostate cancer cells	
	Estimate	Confidence Interval
LNCaP - Ungated	1/11.4	1/10.6 – 1/13.5
LNCaP - Trop2^high^	1/15.9	1/13.3 – 1/18.7
LNCaP - Trop2^low^	1/18.9	1/16.1 – 1/22.3
22Rv1 - Ungated	1/8.3	1/4.9 – 1/13.9
22Rv1 - Trop2^high^	1/7.6	1/4.4 – 1/12.9
22Rv1 - Trop2^low^	1/11.2	1/6.6 – 1/19

In order to analyze their response to therapeutic agents *in vitro*, TROP2^high,^ TROP2^low^ and ungated LNCaP cells were sorted by FACS and treated for 5 days with docetaxel alone or in combination with 10μM flutamide. Cell viability was assessed immediately after treatment cessation (Figure 3C) or 7 days after withdrawal of docetaxel (Figure 3D).

The number of residual live cells was significantly lower after a 7-day treatment withdrawal compared to straight after treatment cessation regardless of the cell population (Figure 3C–3D). Since docetaxel was the only compound present under all conditions this indicates that docetaxel-induced cell death continued well after the removal of this compound from the culture medium.

Dose-response curve analysis indicated that docetaxel decreased the viability of TROP2^high^, TROP^low^ and ungated LNCaP cells with similar IC_50_ (Supplementary Figure S3), suggesting that cytotoxic treatment kills these different cell populations with a similar potency.

However, when we analyzed the maximal effect of docetaxel (defined here as the mean percentage of viable cells that remain after exposure to the three highest concentrations of docetaxel – 1nM, 10nM and 100nM), we found that a significantly higher percentage of TROP2^high^ cells remained alive compared to TROP2^low^ cells, both a the end of the high dose docetaxel treatment (DTX) or following a 7-day relapse (Figure 3C–3D). Our finding demonstrates that docetaxel kills TROP2^high^ cells with a lower maximal efficacy compared to TROP2^low^ cells. Similar results were also found for androgen-sensitive 22Rv1 cells (Supplementary Figures S3 and S4).

When androgen-sensitive cells were incubated with docetaxel in the presence of flutamide (DTX + FLT), TROP2^high^ 22Rv1 cells were again able to recover more efficiently from exposure to high docetaxel concentrations (Supplementary Figure S4), while this was not detected in LNCaP cells (Figure 3D), where the recovery of all cell population was similar after a 7-day treatment withdrawal (Figure 3D).

### Enhanced self-renewal in TROP2^high^ cells from androgen-independent prostate cancer cell lines

**Figure 4:**
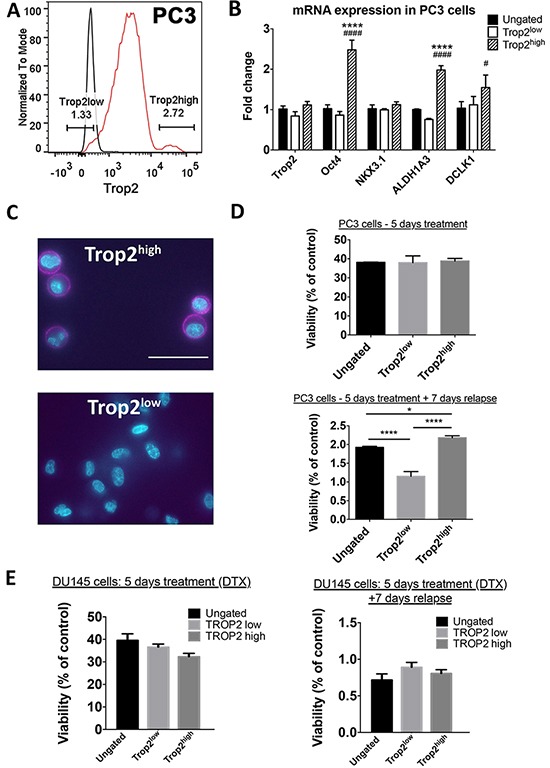
**A.** FACS profile of PC3 cells according to their surface TROP2 expression level. Black line: cells were stained mouse isotype control; red line: cells were stained with TROP2 antibody. **B.** RT-qPCR quantitation of *TACSTD2* (TROP2), *POU5F1* (Oct4), *NKX3.1*, *ALDH1A3* and *DCLK1* mRNA expression. **C.** IF staining for TROP2 on PC3 cells sorted as in (A). Scale bar = 50μM. **D.** Percentage of viable TROP2^high,^ TROP2^low^ and ungated PC3 cells at the end of a 5-day treatment with docetaxel (top) or following a 7-day recovery phase in docetaxel-free medium (bottom). Data represents the mean percentage of surviving cells after exposure to the three highest concentrations of docetaxel (1nM, 10nM, 100nM). **E.** Percentage of viable TROP2^high,^ TROP2^low^ and ungated PC3 cells at the end of a 5-day treatment with docetaxel (left) or following a 7-day recovery phase in docetaxel-free medium (right). Data represents the mean percentage of surviving cells after exposure to the three highest concentrations of docetaxel (1nM, 10nM, 100nM). In D and E: *, P < 0.05, ***, P < 0.001, one-way ANOVA with Bonferroni post-hoc, n = 3);

To determine whether high TROP2 expression also characterizes a sub-population of cells with enhanced resistance to high dose chemotherapy in androgen-independent cells, we then characterized the TROP2^high^ population in the PC3 and DU145 cell lines. Approximately 2.7% of PC3 cells were found to highly express extracellular TROP2 (Figure 4). TROP2 mRNA was not enriched in TROP2^high^ PC3 cells compared to other populations (Figure 4B), but we found that the amount of TROP2 targeted to the extracellular membrane was indeed much higher in TROP2^high^ cells than in any other populations (Figure 4C). Quantification using ELDA indicated that TROP2^high^ PC3 cells had higher spheroid forming capacity than ungated cells and TROP2^low^ cells (Table 2 and Supplementary Figure S5). Similar results were obtained with TROP2^high^ cells isolated from the androgen-independent cell line DU145 (Table 2 and Supplementary Figure S5). In addition, TROP2^high^ PC3 cells showed high mRNA levels of cancer stem cell markers such as OCT4, ALDH1A3 and DCLK1.

**Table 2:** Sphere-forming efficiency of sorted PC3 and DU145 cells sorted based on extracellular Trop2 expression level, as calculated using the ELDA webtool

Cell population	Sphere-forming frequency of androgen-independent cells	
	Estimate	Confidence Interval
PC3 - Ungated	1/51.5^#^	1/43.8 – 1/60.4
PC3 - Trop2^high^	1/18.2^*^^#^	1/15.4 – 1/21.4
PC3 - Trop2^low^	1/127.7^*^	1/104.5 – 1/156.0
DU145 - Ungated	1/25.2	1/14.5 – 1.39.5
DU145 - Trop2^high^	1/5.6^*^^#^	1/3.3 – 1/9.4
DU145 - Trop2^low^	1/18.2	1/11.1 – 1/30.1

* significantly different from ungated population (P<0.0001);

# significantly different from Trop2^low^ cells (P<0.0001, Pearson Chi-square test).

Since the expression of TROP2 was previously shown to overlap with ALDH activity - a putative TIC marker of clinical relevance [18], we performed additional ELDAs to assess whether the high self-renewal ability of TROP2^high^ PC3 cells was associated with their ALDH activity. However, we found that extracellular TROP2 expression predicted the spheroid-forming frequency of PC3 sub-populations irrespective of their ALDH activity. Indeed, TROP2^high^ALDH^low^ and TROP2^high^ALDH^high^ cells showed a similarly high sphere-forming frequency, indicating that their self-renewal ability is 5 to 10 times higher compared to all other cell sub-populations within this cell line (Table 3). This suggests that TROP2 expression is a better predictor of tumor-initiating properties than ALDH in these androgen-independent cells.

**Table 3:** spheroid-forming efficiency of sorted PC3 cells according to their surface Trop2 expression and ALDH activity, as calculated using the ELDA webtool

Cell population	Sphere-forming frequency	
	Estimate	Confidence Interval
Ungated	1/62.6	1/49.5 - 1/79.1
ALDH^high^Trop2^high^	1/16.8^*^	1/13.3 – 1/21.2
ALDH^high^Trop2^medium^	1/129.3	1/97.3 – 1/171.7
ALDH^low^Trop2^high^	1/13.3^*^	1/10.5 – 1/16.9
ALDH^low^Trop2^medium^	1/74.8	1/58.7 – 1/95.3
ALDH^low^Trop2^low^	1/182.0	1/131.9 – 1/251.2

* significantly different from Trop2^medium^ and Trop2^low^ populations (P<0.0001, Pearson Chi-square test).

To investigate whether TROP2 expression is also associated with differential chemoresistance abilities in androgen-independent cells, we exposed PC3 and DU145 cells to docetaxel for 5 days, followed or not by a 7-day post-treatment withdrawal. Similar to what was found for androgen-sensitive cells, significantly lower numbers of viable cells were detected after post-treatment withdrawal compared to right after treatment cessation (Figure 4). No significant survival difference in cell viability between TROP2^high^ and other cell populations was observed immediately after a 5-day treatment with the chemotherapeutic drug docetaxel for either of the two cell lines (Figure 4D–4E and Supplementary Figure S6–S7). Seven days after withdrawal of this compound, the proportion of surviving TROP2^low^ PC3 cells was lower than those in ungated and TROP2^high^ PC3 cells (Figure 4D and Supplementary Figure S6), but this was not the case for DU145 cells (Figure 4E and Supplementary Figure S7), suggesting that this may not be an overall characteristic of androgen-independent TROP2^high^ cells. Taken together, these results indicate that the TROP2^high^ population displays a stronger self-renewal potential than other cells in androgen-independent prostate cancer cells, but does not identify a cell sub-population with enhanced chemo-resistance ability.

### A functional role for TROP2 in LNCaP cell post-treatment recovery in vitro

**Figure 5:**
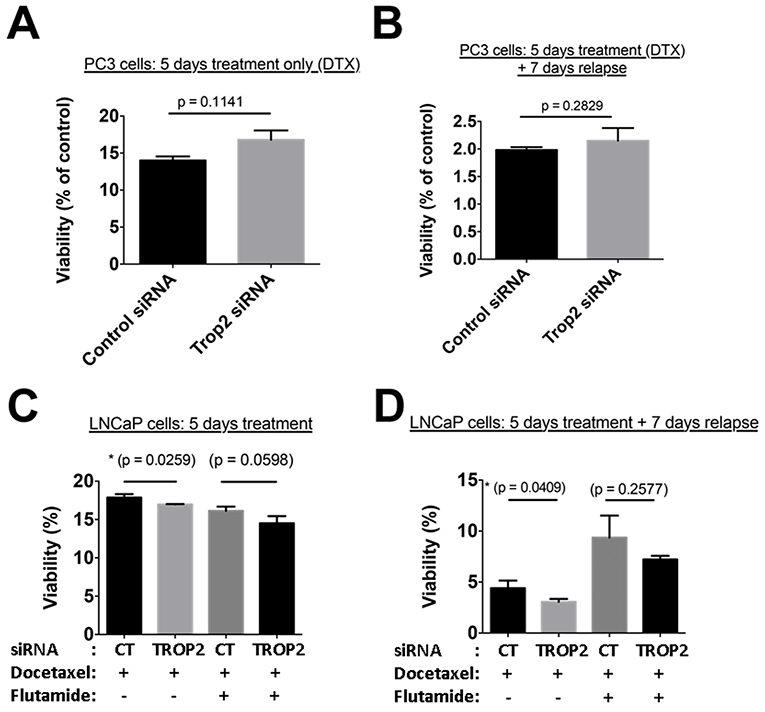
**A–B.** Percentage of viable PC3 cells transfected with control or TROP2-specific siRNA at the end of a 5-day treatment with docetaxel (A) or following a 7-day recovery phase in docetaxel-free medium (B). **C–D.** Percentage of viable LNCaP cells transfected with control or TROP2-specific siRNA and with docetaxel alone or in combination with flutamide (as indicated), at the end of a 5-day treatment (C) or following a 7-day recovery phase in docetaxel-free medium (D). P values are indicated (t-test, n = 3).

To investigate whether TROP2 has a functional role in treatment resistance, androgen-independent PC3 cells and androgen-sensitive LNCaP cells were transfected with TROP2 siRNA to selectively down-regulate TROP2 expression. The down-regulation of extracellular membrane TROP2 expression compared to cells transfected with a control siRNA was confirmed by flow cytometry and baseline growth was unaffected in cells transfected with TROP2 siRNAs (Supplementary Figure S8). No difference in cell viability was observed between control and TROP2 siRNA transfected PC3 cells immediately after 5-day docetaxel treatment (Figure 5A) or 7 days after docetaxel withdrawal from the culture medium (Figure 5B).

Treatment of LNCaP cells with docetaxel for 5 days affected the viability of both control and TROP2 siRNA-transfected cells with a similar IC_50_, although survival in the presence of high docetaxel concentrations (with or without flutamide) was slightly reduced in TROP2 siRNA-transfected cells compared to controls (Figure 5C). When LNCaP cells were maintained in culture for a further 7 days after withdrawal of high docetaxel concentrations, cell recovery was significantly impaired in populations expressing TROP2 siRNA compared to controls (Figure 5D). A similar trend, albeit non-significant, was observed in cells recovering from the combination (docetaxel + flutamide) treatment. (Figure 5D).

These findings suggest that TROP2 may play a role in the ability of androgen-sensitive, but not of androgen-independent prostate cancer cells, to avoid cell death and/or to recover from treatment following exposure to high concentrations of chemotherapeutics.

## DISCUSSION

In the present study we demonstrate that high expression of cell-surface TROP2 is a marker of, and plays a functional role in the ability of androgen-sensitive prostate cancer cells to recover from docetaxel-based chemotherapy, and that high TROP2 expression is a marker of poor prognosis in patients with low-grade prostate tumors. In androgen-independent prostate cancer cells however, TROP2 does not appear to mark cells with enhanced treatment resistance ability but rather enriches cell sub-populations with high self-renewal potential, which represent a characteristic of cancer stem cells and progenitor cells.

We first found that androgen-sensitive LNCaP and 22Rv1 cells that exhibit high cell-surface TROP2 expression recover more efficiently than other cell sub-populations following a 5-day treatment with high concentrations of docetaxel, as seen after a 7-day withdrawal phase in the absence of treatment. This result provided a cellular rationale in support of our *in vivo* results demonstrating the enriched presence of TROP2 mRNA and membrane TROP2 immunostaining in tumors that recur following treatment with docetaxel alone or in combination with flutamide. Highlighting the clinical significance of our findings, these results are in accordance with our observation that high TROP2 expression correlates with poor prognosis in cohorts of prostate cancer patients, particularly in patients with low (Gleason 6) grade tumors. Extracellular expression of TROP2 has been found to correlate with poor prognosis in other cancers including breast [19], gastric [20] as well as gliomas [21], suggesting that TROP may also represent a functional marker for cell sub-populations with enhanced ability to avoid cell death and/or to recover from treatment in these cancers.

Flutamide weakly but significantly slowed the growth of LNCaP xenografts *in vivo* when used alone, a result seemingly at odds with a reports demonstrating it can act as a partial agonist on cells carrying a mutated version of the androgen receptor, such as LNCaP cells [17, 22]. However, the agonist activity of flutamide is most readily measured in the absence of endogenous ligands, and we suggest that competition of flutamide with endogenous androgens such as di-hydro testosterone for receptor binding may have contributed to this apparent growth reduction, as flutamide is not as potent an agonist as DHT [23]. Similar reversion of DHT-induced LNCaP cell growth by flutamide has been shown to occur *in vitro* [24]. Newer generation antagonists such as bicalutamide and enzalutamide, which are effective against the mutated receptor and do not have agonistic activity against LNCaP cells [25, 26], would most likely produce a more robust antitumour effect.

Because tumor-initiating/cancer stem cells have been suspected to play a role in androgen ablation and chemotherapy resistance as well as post-treatment prostate cancer relapse [27], we also analyzed whether the enhanced treatment recovery displayed by TROP2^high^ cells was associated with high self-renewal ability. However we did not find any difference between the self-renewal ability of TROP2^high^, TROP2^low^ and ungated cells from the LNCaP and 22Rv1 cell lines, suggesting that the androgen-sensitive cells that emerge post-treatment may not necessarily bear intrinsic cancer stem cell characteristics. Accordingly the expression of candidate prostate cancer stem cell markers such as CD49f [11] and CD44 [28](not shown) was not found to be elevated in TROP2^high^ LNCaP cells *in vitro* or in post-treatment recurring LNCaP xenografts *in vivo*.

In addition, we found that transfecting cells with TROP2 siRNA significantly reduced the percentage of viable LNCaP cells 7 days after docetaxel withdrawal, suggesting that the TROP2 glycoprotein itself may play an active role in the recovery process of these cells after chemotherapy androgen ablation and treatments. Down-regulation of TROP2 was previously shown to inhibit chemotherapy-induced keratinocyte apoptosis [29] and a TROP2-targeting approach demonstrated robust antitumor activity in triple-negative breast cancer [30]. Our current findings suggest that targeting TROP2 in combination with chemotherapy may improve treatment efficiency in androgen-sensitive prostate tumors.

In contrast, TROP2 siRNA transfection failed to alter PC3 cell viability in response to docetaxel treatment, in accordance with the lack of observed capacity for TROP2^high^ cells to recover more efficiently after treatment in androgen-independent cells. This results suggests that the apparent dependency of androgen-sensitive cells upon TROP2 to improve recovery from cytotoxic treatment may be lost during progression towards the androgen-independent stage, at which point prostate tumors are reported to be more aggressive and chemotherapies such as docetaxel are known to be poorly effective overall [3]. Nevertheless, we found that androgen-independent PC3 and DU145 cells expressing high levels of extracellular TROP2 have higher sphere-forming efficiency than TROP2^low^ and ungated cells. The capacity to self-renew is a critical property of tumor-initiating cells, fuelling the long-term maintenance of malignant clones [31]. Under spheroid culture conditions, which are known to promote self-renewal in various cancers [32], TROP2^high^ PC3 cells consistently generated TROP2^high^ cells only (Supplementary Figure S9), highlighting their capability to self-renew without differentiating and thus demonstrating that the phenotype of these self-renewing cells is stable under such conditions. In contrast, under the same self-renewal-promoting culture conditions, TROP2^low^ PC3 cells were able to produce a small population of TROP2^high^ cells (Supplementary Figure S9), corroborating prior demonstrations of plastic transition from non-stem to stem-like phenotypes in non-neoplasic and cancer cells [33]. These results suggest that TROP2^high^ PC3 cells display a tumor-initiating cell-like phenotype, complementing previous reports that identified TROP2 as a promoter of stemness in the normal prostate epithelium [11, 12] but linking it to an androgen-independent phenotype. Our results show that in PC3 cells, self-renewability is associated with extracellular expression of TROP2 independently from ALDH activity. Cells with high ALDH activity have been proposed to have tumor-initiating activity in several solid cancers including colon, breast, liver, ovarian and pancreatic cancer [34], but in the present study ALDH activity-based selection did not enrich for PC3 cells with enhanced self-renewal in vitro (Table 3) or enhanced tumor-initiating ability in vivo (Supplementary Table S1).

In summary, while extracellular TROP2 overexpression had previously been reported to enhance prostate cancer cell migration and metastatic ability [35] and to correlate with prostate cancer aggressiveness [13], results of the present study provide the first demonstration that high membrane TROP2 expression characterizes different cell subpopulations in androgen-sensitive and androgen-independent prostate cancer cells. We identified androgen-dependent TROP2^high^ cells as a population of cells that drives post-treatment re-growth, while high membrane TROP2 expression represents a hallmark of self-renewing TICs in aggressive androgen-independent tumor cells. Thus, our results suggest that extracellular TROP2 expression could serve as a prognostic indicator of treatment outcome in prostate cancer, and that targeting cancer cells with high surface expression of TROP2 may be an effective way to prevent progression towards castration resistant prostate cancer.

## MATERIALS AND METHODS

### Immunohistochemistry

#### Human prostate cancer tissue sections

Slides of human prostate cancer tissues were obtained and stained under ethics agreement HREC2012.275 (Royal Melbourne Hospital, Melbourne, Australia). Sections were dewaxed, rehydrated and antigen retrieval was performed for 15 min in Citrate buffer (pH 6.10). Endogenous peroxidase activity was blocked using 3% H2O2 in MetOH for 20min at room temperature. Slides were incubated for 2h in blocking buffer alone, followed by an overnight incubation at 4C in blocking buffer containing anti-TROP2 or isotype antibodies (BD Bioscience 551317, 1/500). Biotinylated anti-mouse secondary antibody (1/500, AbCAM) was incubated for 30 min at room temperature, followed by Tyramide Signal Amplification (TSA)(Perkin Elmer, Melbourne, Australia), revelation with diaminobenzidine (DAB) detection kit (AbCAM, Melbourne, Australia), Ethanol/Xylene dehydration and mounting. The percentage of cells considered as positive for TROP2 staining (cf example in Figure 1A) was calculated after counting positive and total cell numbers in 5 randomly chosen fields of approximately 100 tumor cells.

### Tumor xenografts

Slide-mounted cryo-cut tumor sections were fixed with 4% paraformaldehyde for 10 minutes and stained with mouse anti-human TROP2 antibody (BD Bioscience 551317, 1/200) overnight at 4°C followed by incubation with fluorescein horse anti-mouse IgG (Vector laboratories, 1/200) for 1 hour at room temperature. Hoechst 33342 (Invitrogen) was used to stain nuclei. Fluorescent images were taken with a Nikon digital camera attached to a Nikon A1R confocal microscope (Nikon).

### SurvExpress data analysis

The correlation between TROP2 expression and disease outcome was analyzed using the SurvExpress bioresource [14] (http://bioinformatica.mty.itesm.mx:8080/Biomatec/SurvivaX.jsp). Selected datasets were originally from the Gene Expression Omnibus (GSE16560, 281 patients [16], and GSE40272, 98 patients [15]). Selection criteria included minimal patient number per study (>80), presence of *TACSTD2* (TROP2)-specific probes, and availability of survival and/or recurrence-free survival data. Presented data summarizes TROP2 expression levels (median, 5-95 percentile) and Kaplan-Meier analysis (Hazard ratio) of survival or recurrence-free survival as indicated in high and low risk patient subgroups. Optimized risk scores were determined as described in [14].

### LNCaP xenografts

NOD/SCID mice were obtained from the Monash Animal Research Platform and ethical approval for *in vivo* experiments was obtained from the Monash University Standing Committee on Ethics in Animal Experimentation (Ethics numbers: VCPA.2010.33 and MIPS.2012.15).

LNCaP cells (1×10^6^) were injected subcutaneously into the left flank of NOD/SCID mice and treatments were initiated once tumors reached a volume of 100mm^3^. The timeline for the experiment is provided in Supplementary Figure S1, with the different treatment groups as follows: The control group received a slow-releasing pellet containing placebo and an intra-venous injection of vehicle; the flutamide group (chemical androgen ablation) received 33mg/kg/day flutamide and an intra-venous injection of vehicle once a week for four weeks; the docetaxel chemotherapy group received a placebo pellet and an intra-venous injection of 10mg/kg docetaxel once a week for four weeks; The Combined group (flutamide + docetaxel) received a combination of the flutamide and docetaxel doses indicated above. Flutamide treatment was performed by implanting a slow-release flutamide pellet on the lateral side of the neck of the mouse. An incision equal in diameter to that of the pellet (approximately 6mm) was made using sterile surgical scissors. A pocket horizontal to the cut was made using blunt forceps about 2cm beyond the incision site. A slow-releasing flutamide pellet (100mg for 90-days releasing 33mg/kg/day, Innovative Research of America) or placebo pellet (Innovative Research of America) was placed into the pocket with forceps and angled to the right on the lateral side of the neck between the ear and shoulder where there is maximal space between the skin and muscle. The incision site was then sealed with 9mm BBL™ AUTOCLIP wound clips (BD Diagnostics).

Stock solutions of 20mg/ml docetaxel (Tocris) were prepared in 100% ethanol. A final dilution was made just prior to injection in a vehicle of Tween 80 (Sigma) and 5% dextrose (5/5/90 vol/vol/vol).

Mice treated with docetaxel or combination therapy were culled when tumor relapse had occurred, defined by an increase in tumor size in two consecutive measurements following the end of treatment. Due to slight variations in treatment response among individual mice, individual animals were culled at slightly different time points. In control and androgen ablation groups, mice were culled 49 days after the beginning of treatment when the first mouse in the control group had to be culled when tumors had grown to a diameter of >12mm, as per ethical requirements (n=5).

### Cell culture

Human prostate cancer cell lines PC3 (CRL-1435) and LNCaP (CRL-1740), DU145 (HTB-81) were obtained from American Type Culture Collection (ATCC) and cultured in Ham's F-12K (Invitrogen) or RPMI medium 1640 (Invitrogen). The 22Rv1 cell line (CRL-2505) was obtained from Prof. Martin Lackmann (Department of Biochemistry, Monash University, Melbourne, Australia). Media for all cell lines was supplemented with 10% Fetal Bovine Serum (FBS) (Invitrogen 10100-147). The usual androgen concentration within FBS (0.1–1 nM Di Hydro Testosterone) is sufficient to efficiently maintain androgen-sensitive cells such as LNCAP and 22RV1 [36].

For spheroid culture, cells were grown in serum-free DMEM/F-12 medium (Invitrogen 10565) supplemented with 1% N2 (Invitrogen 17502-048), 2% B27 (Invitrogen 17504-044), 20 ng/mL human epidermal growth factor (EGF Invitrogen PHG0311), 20ng/mL human fibroblast growth factor-basic (FGF-b Invitrogen PHG0026) and 100 units of Penicillin-Streptomycin (Invitrogen) in ultra-low attachment plates (Costar).

### FACS analysis and sorting

Cells were stained with Mouse anti-human TROP2 antibody (BD Bioscience 551317, 1/200) in antibody staining buffer (0.9% (w/v), sodium azide and 2% FBS in PBS) for 1 hour. Following three washes in PBS, Alexa Fluor® 647 Goat Anti-Mouse IgG secondary antibody (Invitrogen A21236, diluted 1 in 1000 in antibody staining buffer) was incubated for one hour at 4°C. Stained cells were then washed three times in antibody staining buffer prior to FACS sorting and analyzing.

Cell sorting was performed using a MoFlo®Astrios^TM^ (Beckman Coulter). The gating strategy to define negative populations for ALDH and TROP2 staining was designed so as to include at least 99.9% of cells in the DEAB and isotype negative control samples. A distinct population highly expressing TROP2 was identified and sorted from PC3 cells (~2.75% of total viable cells). The same gating strategy was applied to LNCaP, DU145 and 22Rv1 cells. Since no individualized TROP^high^ population was detected in these cell lines, the TROP2^high^ population was defined as the top 2% of TROP2-expressing cells. FACS analysis was performed using a FACSCanto™ II (BD Science).

### RT-qPCR

mRNA levels for ten genes that were previously reported to act as prostate stem cell or TIC markers or that are involved in cancer progression and metastasis (*CD44*, *ITGA6* (*aka* CD49f), *TACSTD2* (TROP2), *POU5F1* (OCT3-4), *NKX3.1*, *CTNNB1* (beta-catenin), *DCLK1*, *DICER*, *AKT*, *ALDH1A3*) were detected by qPCR in both sorted cells and tumor tissues by RT-qPCR. RNA from both tissue and cells was extracted using the RNeasy Plus Micro kit (Qiagen) following the manufacturer's instructions. Real-time RT-qPCR was carried out with pre-designed human TAQMAN® probes (Invitrogen) with iScript™ One-Step RT-PCR Kit (Bio-Rad) in a CFX96™ Real-Time PCR Detection System (Bio-Rad)., For both PC3 and LNCaP cell lines, glyceraldehyde 3-phosphate dehydrogenase (GAPDH) was used as a housekeeping gene, while, the ribosomal proteins RPLP0 and RPLR30 were used for tumors derived from PC3 and LNCaP xenografts respectively.

### Extreme limiting dilution assay (ELDA)

Cells were sorted and seeded in 3 different concentrations: 10 cells/well, 30 cells/well and 100 cells/well in low-attachment 96-well plates in spheroid culture medium. For each individual experiment, 120 replicates were performed for each concentration of cells. The presence or absence of spheroids (included in the count when size >50μm diameter) in each well was determined at day 14 using a conventional light microscope (Nikon). When run under these conditions, ELDA provides a robust estimate of the tumor-initiating cell frequency, calculated and analyzed using the ELDA webtool (http://bioinf.wehi.edu.au/software/elda)[37].

### *In vitro* toxicity assays

Cells were treated with various concentrations of docetaxel for 5 days and washed three times with PBS at the end of treatment. Cells were then cultured in their ordinary culture medium without docetaxel for 7 days before being tested for cell viability. For the combination treatment, 10μM of flutamide remained in the medium after the withdrawal of docetaxel. Cell viability was assessed using the CellTiter®-Blue cell viability assay (Promega) according to the manufacturer's instructions.

### siRNA transfection

Both PC3 and LNCaP cells were transfected with TROP2-target siRNA (Taqman Silencer® Select siRNA, Thermofisher, https://www.thermofisher.com/order/genome-database/details/sirna/s8366, 50nM final concentration) for 24 hours. Cells were then detached and seeded in 96-well plates 24 hours prior to treatment.

### Data analysis

For each *in vitro* experiment, at least three different passages of cells were used independently in triplicate (n=3). For *in vivo* experiments, 5 mice were used in each experimental group. Data was expressed as Mean ± S.E.M and analyzed using two-way ANOVA with Bonferroni's Post Hoc Test for multiple comparisons using Graph Pad Prism 5.0. A P value < 0.05 was considered statistically significant. For ELDA assay, data was analyzed with the ELDA webtool (http://bioinf.wehi.edu.au/software/elda/index.html) using a Pearson Chi-square test and a P value < 0.05 was considered statistically significant.

## SUPPLEMENTARY FIGURES AND TABLE


