# Supplementary material for: High expression of TROP2 characterizes different cell subpopulations in androgen-sensitive and androgen-independent prostate cancer cells
Source: Oncotarget. 2016 Jun 7;7(28):44492–504. doi: 10.18632/oncotarget.9876 (PMC5190113; doi:10.18632/oncotarget.9876)
Supplement: Supplementary file 1 [file oncotarget-07-44492-s001.pdf]

High expression of TROP2 characterizes different cell subpopulations in androgen-sensitive and androgen-independent prostate cancer cells

SUPPLEMENTARY METHODS

ALDEFLUOR staining

Cells were stained using the ALDEFLUOR assay kit (Stem Cell Technologies) according to the manufacturer's instructions. For negative controls, cells were incubated with the specific ALDH inhibitor diethyaminobenzaldehyde (DEAB). Stained cells were kept at 4°C in ALDEFLUOR assay buffer throughout the sorting process to block the ATP-binding cassette so that cell fluorescence was maintained.

PC3 xenograft-tumor forming assay

50,000, 5,000, 500 or 50 $ALDH^{high}$, $ALDH^{low}$ or ungated cells were suspended in 17.5µl sterile phosphate buffered saline (PBS: mM; NaCl 137, KCl 3, KH2PO4 2, Na2HPO4 8; pH=7.4) and mixed with 17.5µl BD Matrigel™ Basement Membrane Matrix (BD science). Cell suspensions were injected subcutaneously into the left flank of 8-week old NOD/SCID mice. Tumor initiation was considered as confirmed as soon as two consecutive increasing caliper measurements could be taken in previously negative mice. Once measurable tumors were detected, tumor length and width were measured twice a week using digital calipers and volumes were calculated as $width^2 \times length \times 0.5$. Mice were culled when their tumor size reached a diameter of 12mm.

SUPPLEMENTARY FIGURES AND TABLE

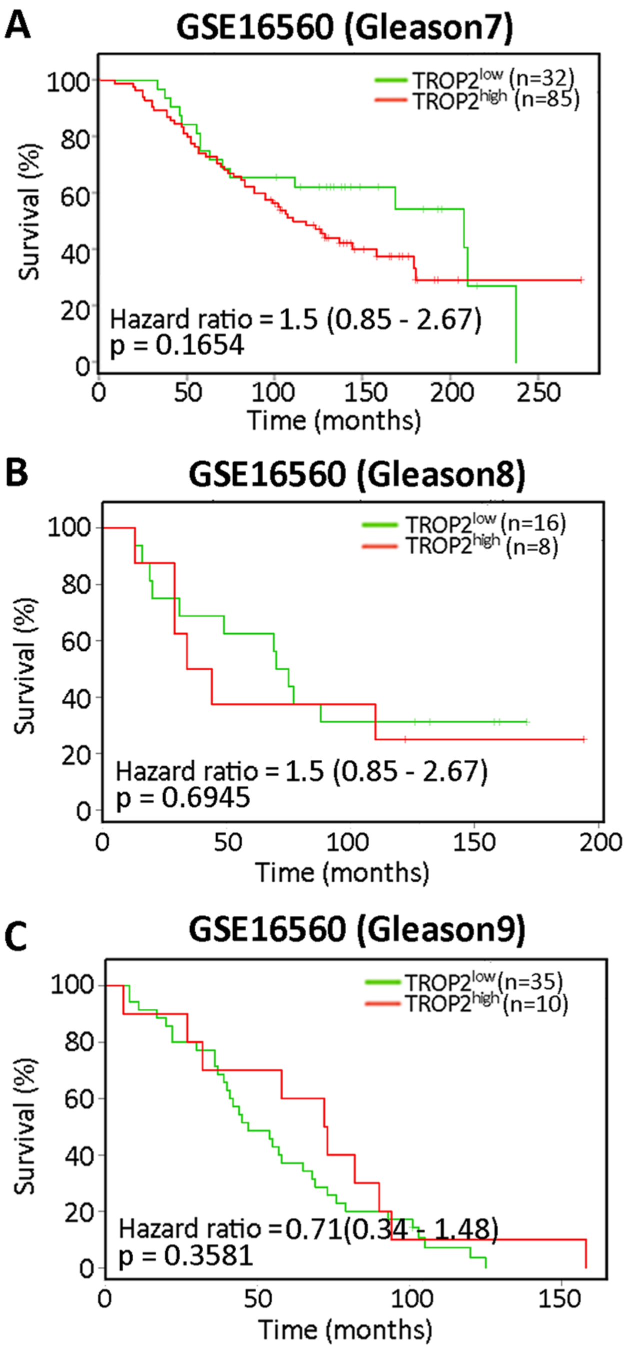

Supplementary Figure S1: Kaplan-Meier curve showing the overall survival of patient carrying Gleason 7 **A.** Gleason 8 **B.** or Gleason 9 **C.** prostate tumors with low or high expression of TROP2 (Data from GSE40272). Hazard ratios with their confidence interval are provided, as well as p values for the log-rank testing equality of survival curves.

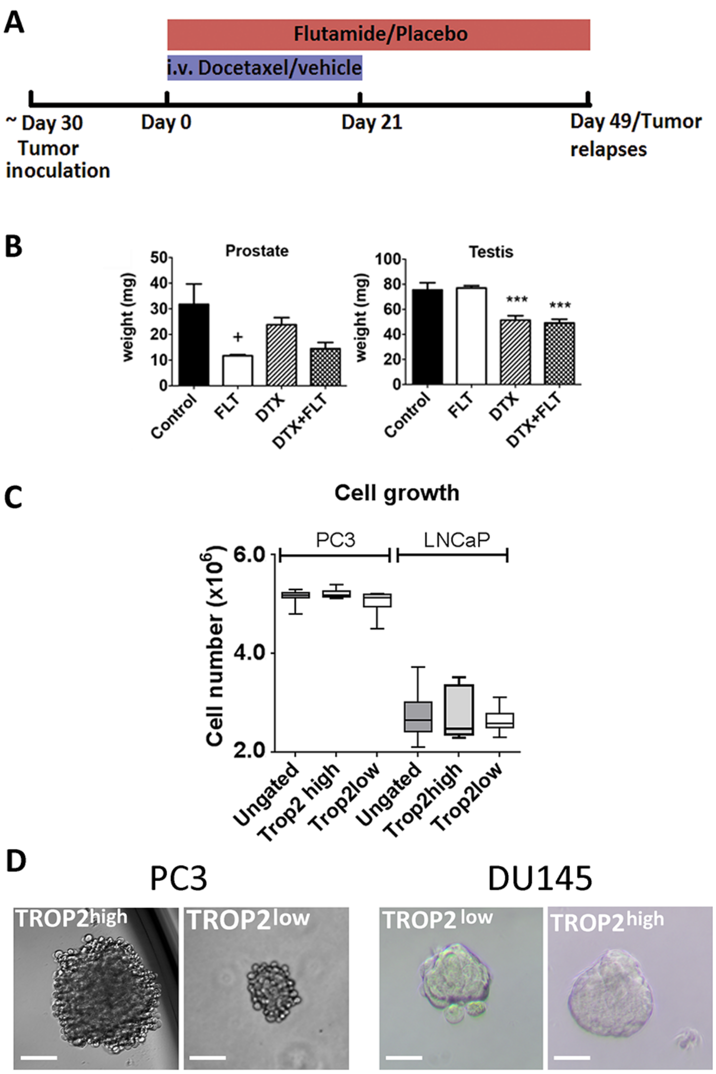

Supplementary Figure S2: A. Schematic outline summarizing LNCaP xenograft and treatment procedures. **B.** Prostate and testis were weighed to ensure the effectiveness of flutamide. +, significantly different from control ($P<0.05$) and *** significantly different from doxetaxel ($P<0.001$) treatment groups. **C.** Box plot representing the baseline number of live cells 72 hours after seeding TROP2^{high,} TROP2^{low} and ungated cells under adherent conditions in the presence of FBS. Cells were manually counted and dead (Trypan Blue-positive) cells were excluded. For each treatment condition, the data is presented as median + 5-95 percentile from 18 measurements across 3 independent experiments. **D.** Representative microphotographs of prostate cancer spheres formed by TROP2^{high} and TROP2^{low} PC3 and DU145 cells as indicated. Magnification bar = 50 µm.

A

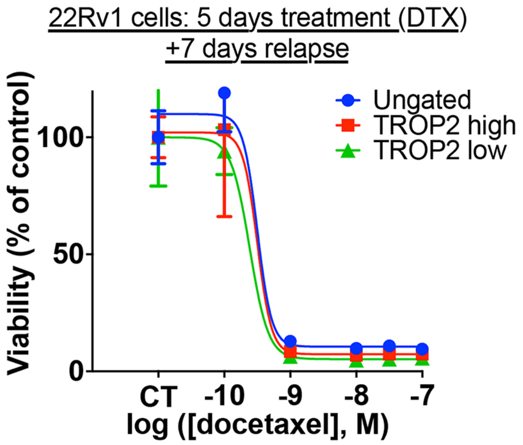

	TROP2 low	TROP2 high	Ungated
Best-fit values			
LogIC50	-9.615	-9.242	-9.140
HillSlope	-3.063	-7.786	-11.06

B

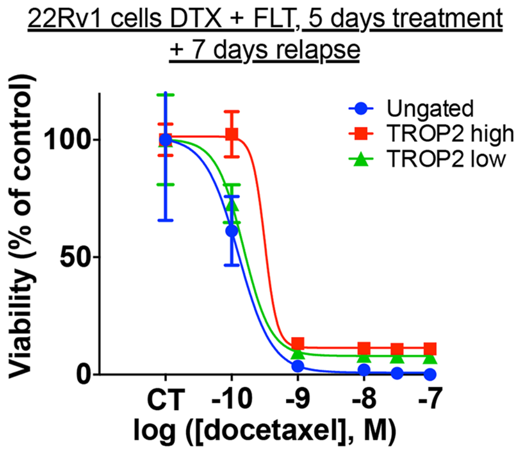

	Ungated	TROP2 high	TROP2 low
Best-fit values			
LogIC50	-9.896	-9.500	-9.822
HillSlope	-1.715	-4.643	-2.100

Supplementary Figure S3: Dose-response toxicity curves of docetaxel on $TROP2^{high}$, $TROP2^{low}$ and ungated 22Rv1 cells, after a 5-day treatment with docetaxel alone (DTX, **A.**) or in combination with flutamide (DTX + FLT, **B.**) followed by a 7-day recovery phase in docetaxel-free medium. $LogIC_{50}$ values and Hill slopes are indicated for all cell populations.

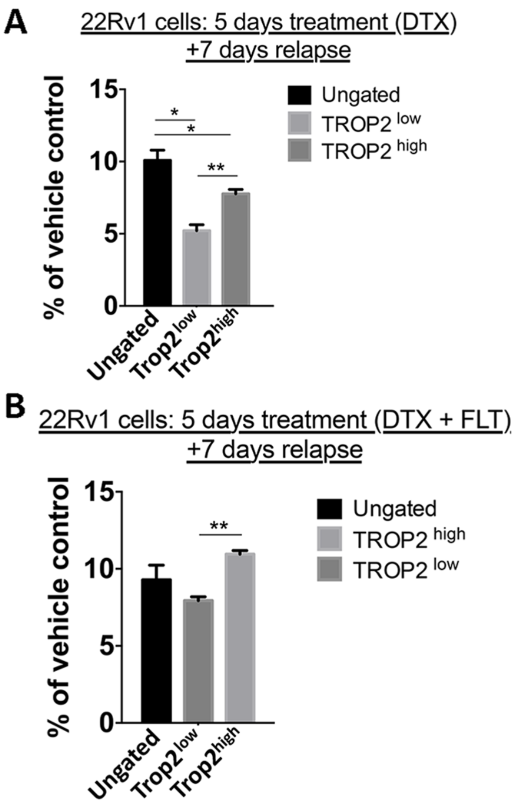

Supplementary Figure S4: Percentage of viable TROP2^{high}, TROP2^{low} and ungated 22Rv1 cells after a 5-day treatment with docetaxel alone (DTX, **A.**) or in combination with flutamide (DTX + FLT, **B.**), followed by a 7-day recovery phase in docetaxel-free medium. Data represents the mean percentage of surviving cells after exposure to the three highest concentrations of docetaxel (1nM, 10nM, 100nM). *, P < 0.05, **, P < 0.01, one-way ANOVA with Bonferroni post-hoc, n = 3.

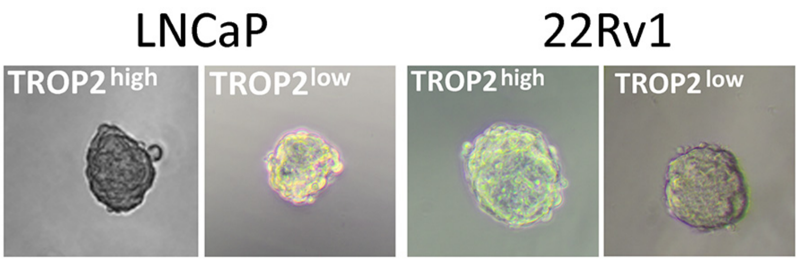

Supplementary Figure S5: Representative microphotographs of prostate cancer spheres formed by TROP2^{high} and TROP2^{low} LNCaP and 22Rv1 cells as indicated. Magnification bar = 50 µm.

A

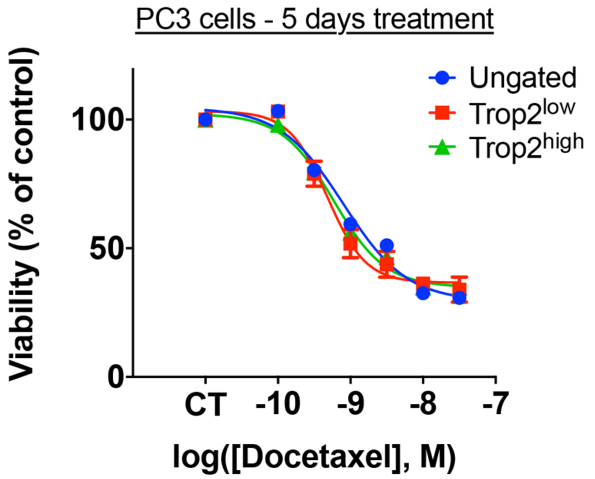

	Ungated	Trop2^{low}	Trop2^{high}
Best-fit values			
LogIC50	-9.094	-9.319	-9.233
HillSlope	-0.9925	-1.637	-1.213

B

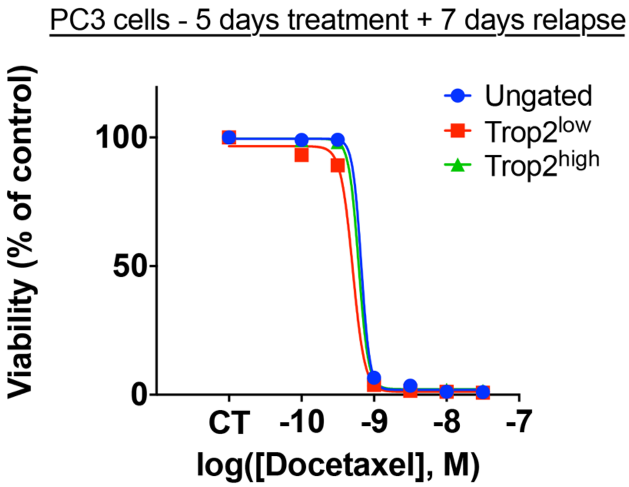

	Ungated	Trop2^{low}	Trop2^{high}
Best-fit values			
LogIC50	-9.177	-9.294	-9.213
HillSlope	-7.305	-5.210	-6.483

Supplementary Figure S6: Dose-response toxicity curves of docetaxel on TROP2^{high}, TROP2^{low} and ungated PC3 cells at the end of a 5-day treatment with docetaxel **A.** or following a 7-day recovery phase in docetaxel-free medium **B.** LogIC$_{50}$ values and Hill slopes are indicated for all cell populations.

A

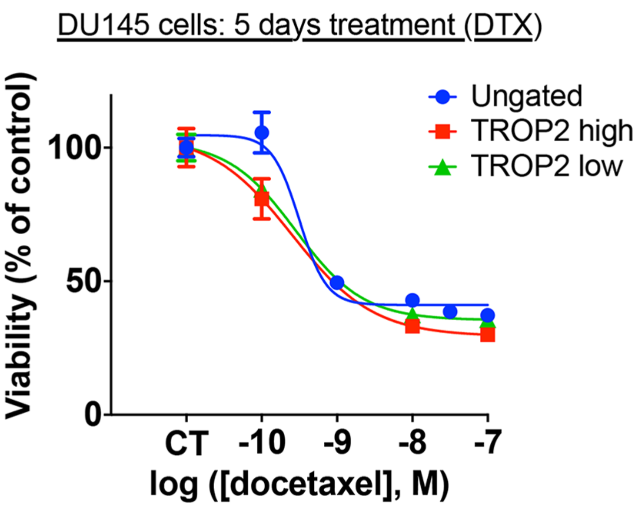

	Ungated	TROP2 high	TROP2 low
Best-fit values			
LogIC50	~ -9.500	-9.596	-9.550
HillSlope	-2.414	-0.8037	-0.9621

B

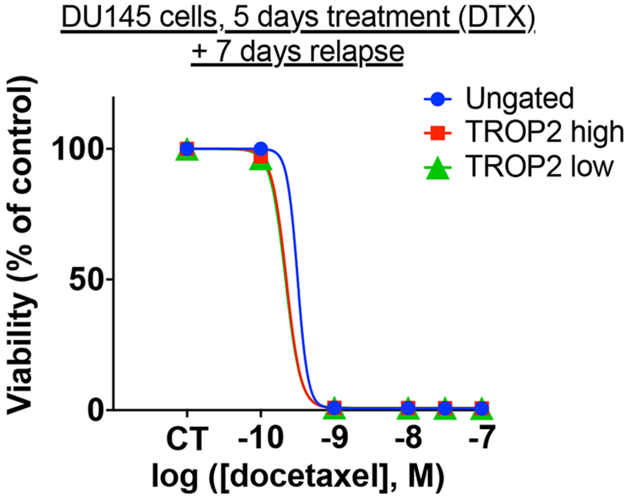

	Ungated	TROP2 high	TROP2 low
Best-fit values			
LogIC50	~ -9.500	-9.654	-9.665
HillSlope	-6.144	-4.417	-4.259

Supplementary Figure S7: Dose-response toxicity curves of docetaxel on TROP2^{high}, TROP2^{low} and ungated DU145 cells at the end of a 5-day treatment with docetaxel **A.** or following a 7-day recovery phase in docetaxel-free medium **B.** LogIC$_{50}$ values and Hill slopes are indicated for all cell populations.

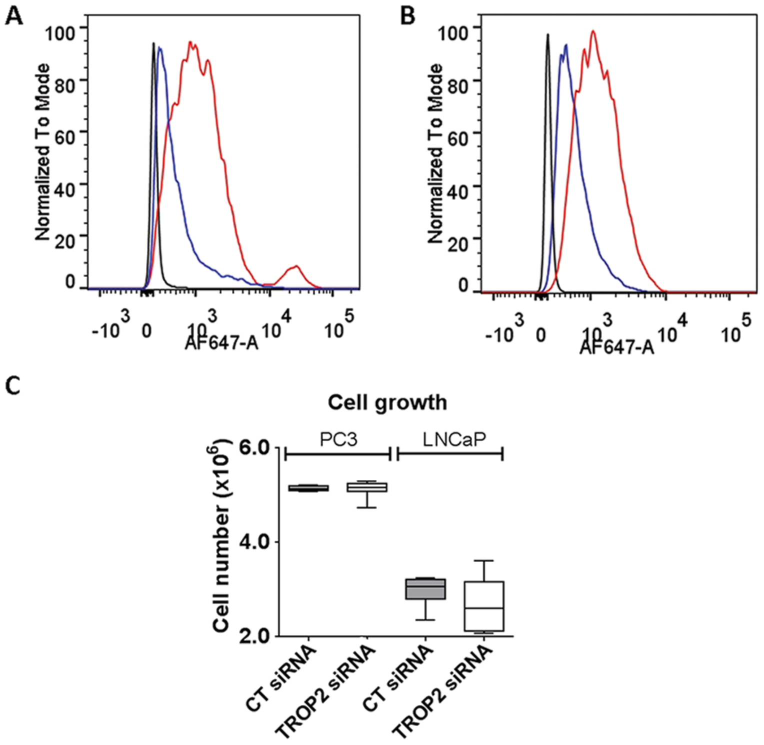

Supplementary Figure S8: FACS analysis of extracellular TROP2 expression in PC3 **A.** and LNCaP **B.** cells 72 hours after transfecting with control siRNA (red line) or TROP2 siRNA (blue line). Black line: cells stained with mouse isotype control. **C.** Box plot representing the baseline number of live cells 72 hours after control or TROP2 siRNA transfection and growth as adherent layers in the presence of FBS. Cells were manually counted and dead (Trypan Blue-positive) cells were excluded. For each treatment condition, the data is presented as median + 5-95 percentile from 18 measurements across 3 independent experiments.

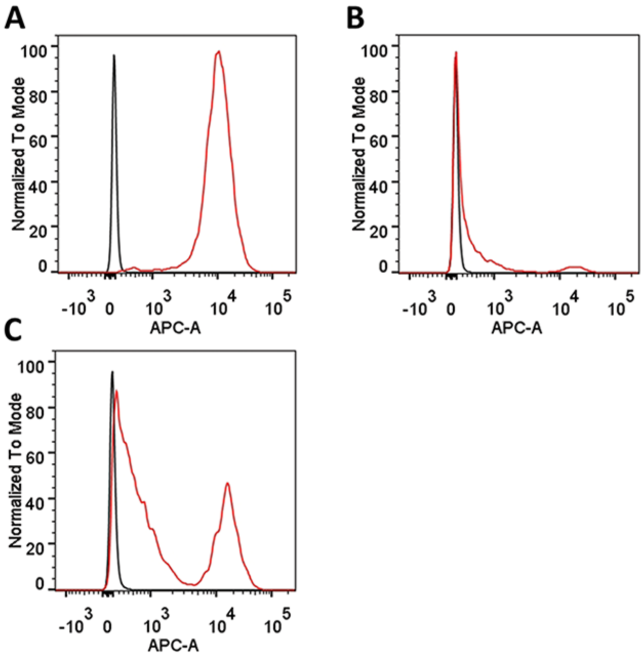

Supplementary Figure S9: FACS analysis of extracellular TROP2 expression in PC3 spheroids 14 days after cell sorting according to TROP2 expression profiles and seeding of purified TROP2^{high} cells **A.** TROP2^{low} cells **B.** or ungated cells **C.**

Supplementary Table S1: Days taken to initiate a measurable tumor in NOD/SCID mice post-injection of 50, 500, 5000 and 500000 PC3 cells with different ALDH activity

Cell number injected	Days taken to initiate a measurable tumor (Mean±SEM)		
	ALDH^{high}	ALDH^{low}	Ungated
50 cells	26.8±2.5	30.4±2.0	31.20±2.1
500 cells	14.2±2.3	22.0±3.8	19.0±2.2
5000 cells	10.4±2.4	13.4±2.0	13.8±1.4
50000 cells	10.8±0.8*	16.4±1.6	14.0±1.2

*, significantly different from injection of the same number of ALDH^{low} cells ($P<0.05$ n=5, one-way repeated measures ANOVA with Bonferroni's Post Hoc Test for multiple comparisons).
